# Identification of biology-based breast cancer types with distinct predictive and prognostic features: role of steroid hormone and HER2 receptor expression in patients treated with neoadjuvant anthracycline/taxane-based chemotherapy

**DOI:** 10.1186/bcr2363

**Published:** 2009-09-16

**Authors:** Silvia Darb-Esfahani, Sibylle Loibl, Berit M Müller, Marc Roller, Carsten Denkert, Martina Komor, Karsten Schlüns, Jens Uwe Blohmer, Jan Budczies, Bernd Gerber, Aurelia Noske, Andreas du Bois, Wilko Weichert, Christian Jackisch, Manfred Dietel, Klaus Richter, Manfred Kaufmann, Gunter von Minckwitz

**Affiliations:** 1 Institute of Pathology, Charité Universitätsmedizin Berlin, Charitéplatz 1, Berlin, 10117, Germany; 2 GBG Forschungs GmbH, Schleussnerstrasse 42, Neu-Isenburg, 63263, Germany; 3 Department of Obstetrics and Gynecology, St. Gertrauden Hospital, Paretzer Str. 12, Berlin, 10713, Germany; 4 Department of Obstetrics and Gynecology, Klinikum Südstadt, Südring 81, Rostock, 18059, Germany; 5 Department of Gynecology and Gynecologic Oncology, Dr. Horst Schmidt Hospital (HSK), Ludwig-Erhard-Str. 100, Wiesbaden, 65199, Germany; 6 Department of Obstetrics and Gynecology, Klinikum Offenbach, Starkenburgring 66, Offenbach am Main, 63069, Germany; 7 Institute of Pathology, Berliner Allee 48, Hannover, 30175, Germany; 8 Department of Obstetrics and Gynecology, Klinikum der Johann Wolofgang Goethe-Universität, Theodor-Stern-Kai 7, Frankfurt am Main, 60590, Germany

## Abstract

**Introduction:**

Reliable predictive and prognostic markers for routine diagnostic purposes are needed for breast cancer patients treated with neoadjuvant chemotherapy. We evaluated protein biomarkers in a cohort of 116 participants of the GeparDuo study on anthracycline/taxane-based neoadjuvant chemotherapy for operable breast cancer to test for associations with pathological complete response (pCR) and disease-free survival (DFS). Particularly, we evaluated if interactions between hormone receptor (HR) and human epidermal growth factor receptor 2 (HER2) expression might lead to a different clinical behavior of HR+/HER2+ co-expressing and HR+/HER2- tumors and whether subgroups of triple negative tumors might be identified by the help of Ki67 labeling index, cytokeratin 5/6 (CK5/6), as well as cyclooxygenase-2 (COX-2), and Y-box binding protein 1 (YB-1) expression.

**Methods:**

Expression analysis was performed using immunohistochemistry and silver-enhanced in situ hybridization on tissue microarrays (TMAs) of pretherapeutic core biopsies.

**Results:**

pCR rates were significantly different between the biology-based tumor types (*P *= 0.044) with HR+/HER2+ and HR-/HER2- tumors having higher pCR rates than HR+/HER2- tumors. Ki67 labeling index, confirmed as significant predictor of pCR in the whole cohort (*P *= 0.001), identified HR-/HER- (triple negative) carcinomas with a higher chance for a pCR (*P *= 0.006). Biology-based tumor type (*P *= 0.046 for HR+/HER2+ vs. HR+/HER2-), Ki67 labeling index (*P *= 0.028), and treatment arm (*P *= 0.036) were independent predictors of pCR in a multivariate model. DFS was different in the biology-based tumor types (*P *< 0.0001) with HR+/HER2- and HR+/HER2+ tumors having the best prognosis and HR-/HER2+ tumors showing the worst outcome. Biology-based tumor type was an independent prognostic factor for DFS in multivariate analysis (*P *< 0.001).

**Conclusions:**

Our data demonstrate that a biology-based breast cancer classification using estrogen receptor (ER), progesterone receptor (PgR), and HER2 bears independent predictive and prognostic potential. The HR+/HER2+ co-expressing carcinomas emerged as a group of tumors with a good response rate to neoadjuvant chemotherapy and a favorable prognosis. HR+/HER2- tumors had a good prognosis irrespective of a pCR, whereas patients with HR-/HER- and HR-/HER+ tumors, especially if they had not achieved a pCR, had an unfavorable prognosis and are in need of additional treatment options.

**Trial registration:**

ClinicalTrials.gov identifier: NCT00793377

## Introduction

Neoadjuvant chemotherapy or preoperative systemic therapy is increasingly considered for patients with operable breast cancer [1,2] as survival rates are similar as in patients receiving standard post-operative chemotherapy and the rate of breast conserving surgery can be significantly increased in patients treated with neoadjuvant chemotherapy [3,4]. One of the main aims of neoadjuvant chemotherapy is to achieve a pathological complete response (pCR; i.e. absence of malignant cells at the tumor site) because pCR has been found to be associated with longer disease-free and overall survival rates [2,5-7]. However, it is not clear if this predictive value is valid for all patients, as a small proportion of patients with pCR still experience distant relapse [8]. In general, pCR rates with classical chemotherapy are rather low and range from 10% to 26% depending on the applied regimes [9]. To date, only a few tumor markers exist for the prediction of pCR, e.g. low tumor differentiation and negative hormone receptor (HR) status [10,11]. Therefore, reliable predictive and prognostic markers are needed for the optimal selection of patients who might benefit from a neoadjuvant chemotherapy, i.e. who have the chance to achieve a pCR and remain disease-free on the long term.

Studies investigating gene expression profiles in breast cancer have defined different breast cancer subclasses that were based on tumor biology-based characteristics [12-15]. Luminal cancers were characterized by the expression of HR, the HER2 cluster showed an over-expression of HER2 and associated genes, and basal-like cancers were negative for HR and HER2 ("triple negative") and express basal cytokeratins as well as the proliferative cluster of genes [12]. Despite the fact that these biology-based tumor types are usually seen as different entities, in clinical practice there is a remarkable overlap between HR and HER2 positive cases. As data from preclinical models suggest an interaction between the HER2 and HR pathways [16,17], we evaluated the hypothesis that these interactions might lead to a different clinical behavior of HR+/HER2+ co-expressing and HR+/HER2- tumors. This might be reflected in a different response to anthracycline/taxane-based neoadjuvant chemotherapy as well as in a different DFS. In addition, it has been suggested that the subgroup of HR-/HER2- (triple negative) carcinomas might constitute a mixture of different biologically and prognostically heterogeneous tumors [18,19]. Therefore, we evaluated the hypothesis that a subclassification of these carcinomas might be possible using Ki-67 proliferation index, cytokeratin 5/6 (CK5/6), cyclooxygenase-2 (COX-2), as well as Y-box binding protein 1 (YB-1) expression, for the latter two a role in breast cancer progression has been demonstrated previously [20,21]. We investigated our hypotheses in a cohort of pretherapeutic core biopsies from the neoadjuvant GeparDuo study, in which patients with operable breast cancer have been treated with either dose-dense doxorubicin plus docetaxel (ddADOC) or conventionally-dosed doxorubicin plus cyclophosphamide followed by docetaxel (AC-DOC) [22].

## Materials and methods

### Study population and histopathological examination

The multicenter randomized prospective neoadjuvant phase III GeparDuo trial (NCT00793377) investigated 913 patients with operable breast cancer (T2-3, N0-2, M0) between June 1999 and September 2001 comparing doxorubicin 50 mg/m^2 ^plus docetaxel 75 mg/m^2 ^every 14 days for four cycles with filgrastim support (ddADOC, n = 451) or four cycles doxorubicin 60 mg/m^2 ^plus cyclophosphamide 600 mg/m^2 ^every 21 days followed by docetaxel 100 mg/m^2 ^every 21 days for four cycles (AC-DOC, n = 453). The trial was conducted in compliance with the Helsinki Declaration. The protocol was reviewed and approved by all responsible local ethics committees. The leading ethics committee was located at the Johann-Wolfgang Goethe University, Frankfurt, Germany (Approval Number: 80/99). Consent of patient, pathologist and investigator to supply tumor material of biopsy and surgery for central pathologic evaluation and examination of predictive factors was available. All patients received tamoxifen simultaneously, irrespective of HR status [22]. The primary endpoint was the incidence of pCR in the breast and axillary nodes (absence of invasive and non-invasive (carcinoma in situ) tumor cells in the surgical specimen including lymph nodes). A statistical analysis using a pCR definition that also includes cases with residual in-situ carcinoma yielded similar results (not shown). For 219 patients tissue from the presurgical biopsy containing more than 30% tumor tissue was available in our tissue bank. These samples were used to construct a tissue microarray. In the statistical evaluation, only cases that could be evaluated at least for ER and HER2 were included (116 cases). Stained slides were digitized by a slide scanner (Mirax Scan, Zeiss, Jena, Germany), and were subsequently evaluated using a custom-made software for whole slide imaging. For clinico-pathological characteristics of our study cohort see additional data file 1. Data according to clinical tumor stage (cT) and clinical lymph node state (cN), patient age, pCR and outcome data were derived from the clinical study database. Punch biopsies were re-evaluated according to tumor histology and grading (Bloom-Richardson modified by Elston and Ellis) [23] by two experienced pathologists (CD and AN). DFS data were available from 105 patients for a median follow-up time without event of 57.6 months.

### Immunohistochemical staining and silver-enhanced in situ hybridization (SISH)

Immunohistochemical staining of tissue microarray slides was performed using the Discovery XT autostainer (Ventana, Tuscon, AZ, USA) according to the manufacturer's instructions. The following antibodies were used: rabbit monoclonal antibody against human ERα (clone SP1, Neomarkers (Lab Vision), Fremont, CA, USA, 1:50); mouse monoclonal antibody against human progesterone receptor PgR (clone PgR 636, Dako, Glostrup, Denmark, 1:50); rabbit polyclonal antibody against human HER2 (HercepTest™ antibody, Dako, 1:500); mouse monoclonal antibody against human Ki67 (clone MIB-1, Dako, 1:50); mouse monoclonal antibody against human CK5/6 (clone D4/16B4, Zymed (Invitrogen), Carlsbad, CA, USA, 1:25). Immunohistochemical staining for YB-1 (Biogenes, Berlin, Germany, 1:1000) and COX-2 (Cayman Chemical Company, Ann Arbor, MI, USA, 1: 5000) was performed manually as described previously [20,21]. SISH analysis was performed on a Benchmark XT autostainer (Ventana) using the INFORM HER2 probe (Ventana) according to the manufacturer's instructions.

ER and PgR immunohistochemistry was scored positive if at least 10% of tumor cell nuclei showed a staining signal. Data on PgR expression were available for 105 cases. HER2 reactivity was assessed according to the ASCO/CAP guidelines [24]: Cases with a uniform intense membranous staining of > 30% of tumor cells (3+) or those with a weak membranous staining (2+) and HER2 amplification in SISH were designated as HER2 positive. For the assessment of the proliferation rate the percentage of tumor cell nuclei positive for Ki67 was estimated (106 cases). According to Petit et al. a cutoff of 20% of stained tumor cell nuclei was used for dichotomization [25]. Any expression of CK5/6 in expression tumor cells was scored as positive (114 cases). For the interpretation of COX-2 and YB-1 staining the immunoreactivity score was used comprising both staining intensity and rate of stained tumor cells, as described previously (101 and 106 cases, respectively) [20]. Immunohistochemistry was evaluated by at least two pathologists who were blinded towards the patients' outcome (SDE and BM).

### Statistical evaluation

Correlation analyses were performed by the use of binary logistic regression analysis, and as indicated by χ^2 ^test. Survival times were compared by Cox regression analysis and the Kaplan-Meier method. *P*-values ≤ 0.05 were considered significant. For statistical procedures, the software packages SPSSv16.0 (Chicago, IL, USA) and GraphPad Prism 5.01 (La Jolla, CA, USA) were used.

## Results

### Distribution of clinico-pathological parameters in the study group

The study cohort was derived from the GeparDuo study that comprised patients with operable breast cancer (cT1-3, cN0-2). Most patients had ductal-invasive breast cancer, a minor subgroup had lobular carcinomas and few patients had cancers of rare histology, which are summarized as "others" in our study (cribriform, metaplastic). Thirteen patients in the study achieved a pCR (11.2%). The rate of patients receiving ddADOC and AC-DOC therapy was similar (*P *> 0.05). The distribution of clinico-pathological parameters was comparable to the full study population [see additional data file 1].

### Immunohistochemical findings in the study cohort

**Figure 1:**
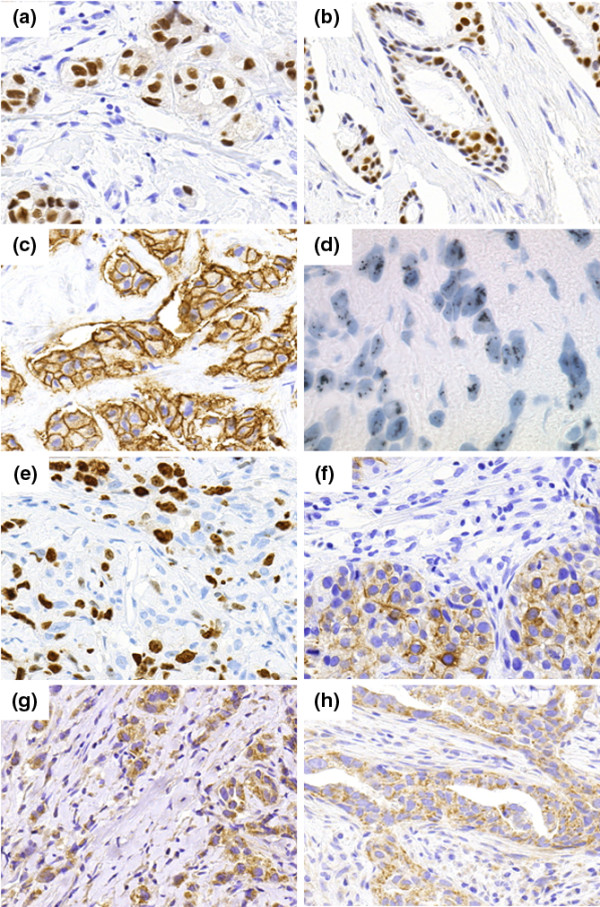
**(a)** Strong expression of ER in a ductal-invasive breast carcinoma. **(b) **Strong expression of PgR in a moderately differentiated breast carcinoma. **(c) **Complete membranous expression of HER2 in a high-grade breast carcinoma (3+). **(d) **Silver-enhanced in situ hybridization (SISH): multiple Her2 gene copies (black dots) in tumor cell nuclei of a Her2-amplified breast carcinoma. **(e) **Nuclear expression of Ki67 in a poorly differentiated breast carcinoma. **(f) **Focal perimembranous expression of CK5/6 in a high grade tumor. **(g) **Moderate cytoplasmic expression of COX-2 in a ductal-invasive breast carcinoma. **(h) **Diffuse cytoplasmic expression of YB-1 in invasive breast cancer nests.

ER was expressed by 63 (54.3%) invasive breast carcinomas (Figure 1A). Fewer tumors were positive for PgR (44 cases, 37.9%, Figure 1B). A co-expression of ER and PgR was found in 37 cases (31.9%). A HER2 over-expression was detected in 27 cases (23.3%, Figure 1C, D). Ki67 (MIB-1) expression ranged from 0 to 90%, median 5%; 85 tumors showed <=20% of positive nuclei (80.2%), 21 tumors had a proliferation rate of > 20% (19.8%; Figure 1E). Seven carcinomas (6.1%) showed evidence of a CK5/6 expression, which was mostly focally (Figure 1F). COX-2 was expressed by 74 tumors (73.3%, Figure 1G), and expression of YB-1 was detected in the cytoplasm of tumor cells in 35 cases (33.0%, Figure 1H).

### Biology-based tumor types according to HR and HER2 status

Data on ER, PgR, and HER2 expression were used to classify breast carcinomas. With a focus on different combinations of these markers, we distinguished four distinct, non-overlapping classes: tumors positive for ER and/or PgR expression and negative for HER2 expression were designated as HR+/HER2- (57 cases, 49.1%). HER2 expressing tumors without HR expression were designated as HR-/HER2+ (13 cases, 11.2%). In case of a co-expression of HR and HER2 the tumor was included in the HR+/HER2+ category (13 cases, 11.2%), while tumors negative for ER, PgR and HER2 were designated as HR-/HER2- (triple negative, 33 cases, 28.4%).

### Association of biology-based tumor types with clinico-pathological parameters as well as YB-1 and COX-2 expression

Biology-based tumor types were significantly associated with tumor grading: While HR+/HER2- positive tumors were most frequently well or moderately differentiated, the proportion of G3 tumors was higher in the HR-/HER2+ as well as HR-/HER- group (*P *= 0.004, χ^2 ^test). Consistent with HER2 being the target of the transcription factor YB-1, HER2 positive tumors (HR-/HER2+ or HR+/HER2+) were most often YB-1 positive (66.7% and 58.3%), as previously reported [26,27], while a YB-1 expression was rare in the HR+/HER2- and HR-/HER- subgroups (25.9% and 25.8%; *P *= 0.018, χ^2 ^test). No significant correlations could be established between COX-2 expression and any of the subgroups investigated. In line with previous reports, CK5/6 expression was mainly found in HR-/HER2- tumors (6/31 cases, 19.4%, *P *= 0.005, χ^2 ^test) [28,29].

### Association of biology-based tumor types with pCR

**Figure 2:**
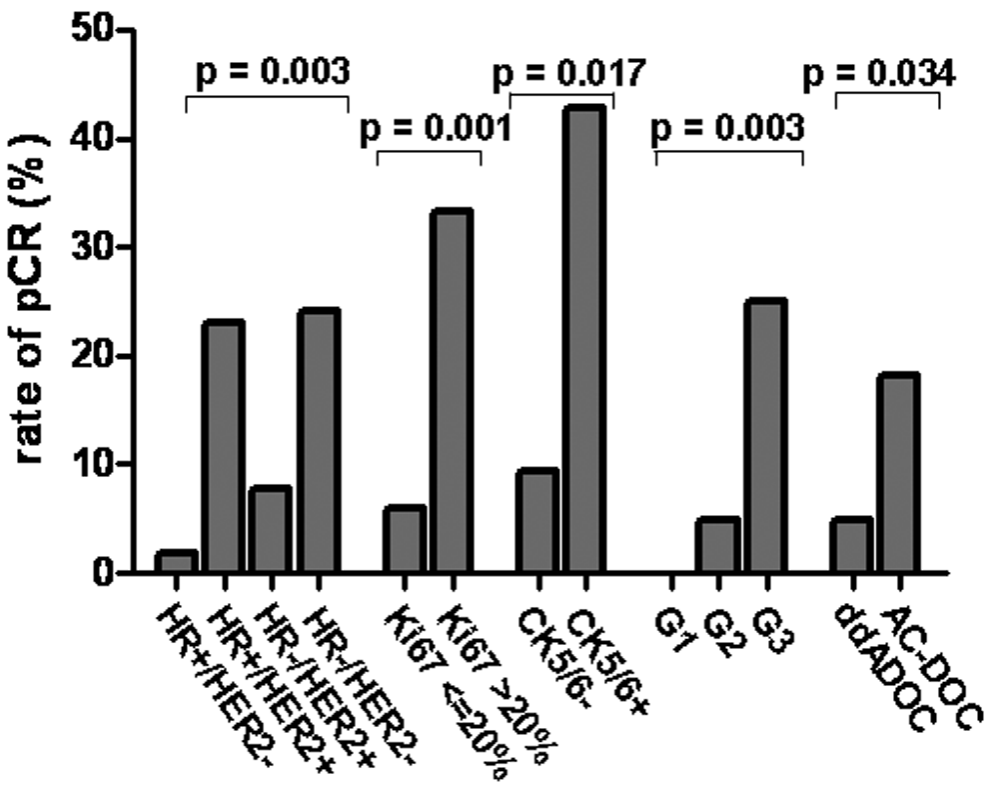
Rate of patients achieving a pCR in dependence of biology-based tumor type (p: likelihood ratio test), Ki67 labeling index, CK5/6 expression, grading, and pre-operative chemotherapy. p: logistic regression.

The pCR rate was significantly different between biology-based tumor types (*P *= 0.044, Figure 2, Table 1). Patients with HR+/HER2- tumors had the lowest pCR rate: only 1 of 57 (1.8%) of those patients experienced a pCR. In contrast, 8 of 33 (24.2%) patients with HR-/HER2- tumors achieved a pCR. The odds ratio for achieving a pCR in the HR-/HER2- group was 17.92 as compared to the HR+/HER2- subgroup (*P *= 0.008). Interestingly, patients with HR+/HER2+ tumors had a similar pCR rate than those with HR-/HER2- tumors (23.1%, hazard ratio HR+/HER2+ vs. HR+/HER2- 16.8, *P *= 0.019, Table 1). In the HR-/HER2+ subgroup, however, the pCR rate was not significantly different from the HR+/HER2- subgroup (7.7%; OR 4.67 *P *= 0.288).

**Table 1:** Correlation with pCR (univariate logistic regression analysis)

	n	events	% pCR	OR	95% CI	p
**age **(per year)	116	13	-	0.96	0.90-1.01	0.128
**biology-based tumor type**	116	13				0.044^a^
HR+/HER2-	57	1	1.8	1.00		
HR+/HER2+	13	3	23.1	16.80	1.59-178.12	0.019
HR-/HER2+	13	1	7.7	4.67	0.27-79.96	0.288
HR-/HER2-	33	8	24.2	17.92	2.13-151.04	0.008
**Ki67**	106	12				
<= 20%	85	5	5.9	1.00		
> 20%	21	7	33.3	8.00	2.22-28.79	0.001
**CK5/6**	114	13				
negative	107	10	9.3	1.00		
positive	7	3	42.9	7.28	1.42-37.22	0.017
**COX-2**	101	12				
negative	27	5	18.5	1.00		
positive	74	7	9.5	0.46	0.13-1.60	0.221
**YB-1**	106	12				
negative	71	8	11.3	1.00		
positive	35	4	11.4	1.02	0.28-3.36	0.980
**therapy arm**	116	13				
ddADOC	61	3	4.9	1.00		
AC-DOC	55	10	18.2	4.30	1.12-16.53	0.034
**histology**	116	13				0.767^a^
ductal-invasive	98	12	12.2	1.00		
lobular	14	0	0.0	0.00	0.00- -	0.999
others	4	1	25.0	2.39	0.23-24.86	0.466
**grade**	116	13				
G1-2	76	3	3.9	1.00		
G3	40	10	25.0	8.11	2.09-31.55	0.003
**cT**	116	27				0.570^a^
cT1	9	2	22.2	1.00		
cT2	87	9	10.3	0.40	0.07-2.25	0.300
cT3	20	2	10.0	0.39	0.05-3.32	0.388
**cN**	116	27				
cN0	79	8	10.1	1.00		
cN1-2	37	5	13.5	1.39	0.42-4.57	0.591

OR: odds ratio; CI: confidence interval;^ a^: overall significance.

### Association of other factors with pCR

The pCR rate was significantly correlated with Ki67 labeling index (*P *= 0.001), CK5/6 expression (*P *= 0.017), tumor grade (*P *= 0.003), and treatment arm (*P *= 0.034) (Table 1 and Figure 2).

### Association with pCR - multivariate analysis

In an exploratory multivariate logistic regression analysis including the significant predictive markers from univariate analysis (biology-based tumor types, Ki67 labeling index, CK5/6 expression, grading, treatment arm; Table 2), the HR+/HER2+ subgroup was independently linked to a higher pCR rate (*P *= 0.046; compared to HR+/HER2-) while the HR-/HER2- group lost its predictive significance. Furthermore, Ki67 staining and treatment arm were of independent predictive value (*P *= 0.028 and *P *= 0.036, Table 2).

**Table 2:** Multivariate analysis (logistic regression and Cox regression analysis)

	correlation with pCR			correlation with survival		
	OR	95% CI	p	hazard ratio	95% CI	p
**biology-based tumor type**			0.258^a^			< 0.0001^a^
HR+/HER2-	1.00			1.00		
HR+/HER2+	14.28	1.05-194.31	0.046	1.88	0.39-9.04	0.432
HR-/HER2+	5.35	0.25-116.83	0.287	16.78	5.75-48.97	< 0.0001
HR-/HER2-	6.01	0.53-67.84	0.147	4.24	1.62-11.12	0.003
**Ki67**						
<= 20%	1.00					
> 20%	10.37	1.29-83.28	0.028	-	-	-
**CK5/6**						
negative	1.00					
positive	0.51	0.04-7.57	0.627	-	-	-
**therapy arm**						
ADoc	1.00					
ACDoc	11.97	1.17-122.16	0.036	-	-	-
**grade**						
G1-2	1.00					
G3	5.59	0.93-33.70	0.061	-	-	-
**ypN**						
cN0				1.00		
cN1-2	-	-	-	2.77	1.24-6.18	0.013
**pCR**						
no pCR				1.00		
pCR	-	-	-	0.18	0.02-1.43	0.104

OR: odds ratio; CI: confidence interval; ^a^: overall significance.

### Association of HR-/HER2-(triple negative) subgroups with pCR

We evaluated the hypothesis that molecular markers could be used to divide the triple-negative tumors into different subgroups wit different clinical outcome. As shown in Table 3, HR-/HER2- tumors could be divided into subgroups with different pCR rates according to their proliferation rate. HR-/HER2- tumors were more likely to respond if they showed an increased Ki67 level: a significantly higher pCR rate was seen in tumors with > 20% positive nuclei (63.6% vs. 0%, *P *< 0.0001, χ^2 ^test), and an increase of 10% of Ki67-labeled tumor cells equaled a hazard ratio of 1.92 (*P *= 0.006, Table 3). Although the pCR rate in HR-/HER2- carcinomas was higher when they expressed CK5/6 (37.5%) compared to 15% for CK5/6 negative tumors, the sub-classification by CK5/6 was not a significant predictor. COX-2, or YB-1 expression were not relevant for response prediction in the HR-/HER2- group.

**Table 3:** Correlation of HR-/HER2- subgroups with pCR (univariate logistic regression analysis)

	n	events	% pCR	OR	95% CI	p
**HR-/HER2- subgroups** **Ki67 **(per 10%)	29	7	-	1.92	1.21-3.04	0.006
**CK5/6**	30	8				
negative	24	5	20.8	1.00		
positive	6	3	50.0	3.80	0.58-24.88	0.127
**COX-2**	26	7				
negative	9	3	33.3	1.00		
positive	17	4	23.5	0.62	0.10-3.66	0.593
**YB-1**	30	7				
negative	22	5	22.7	1.00		
positive	8	2	25.0	1.13	0.17-7.47	0.896

OR: odds ratio; CI: confidence interval.

### Association of biology-based tumor types with DFS

**Figure 3:**
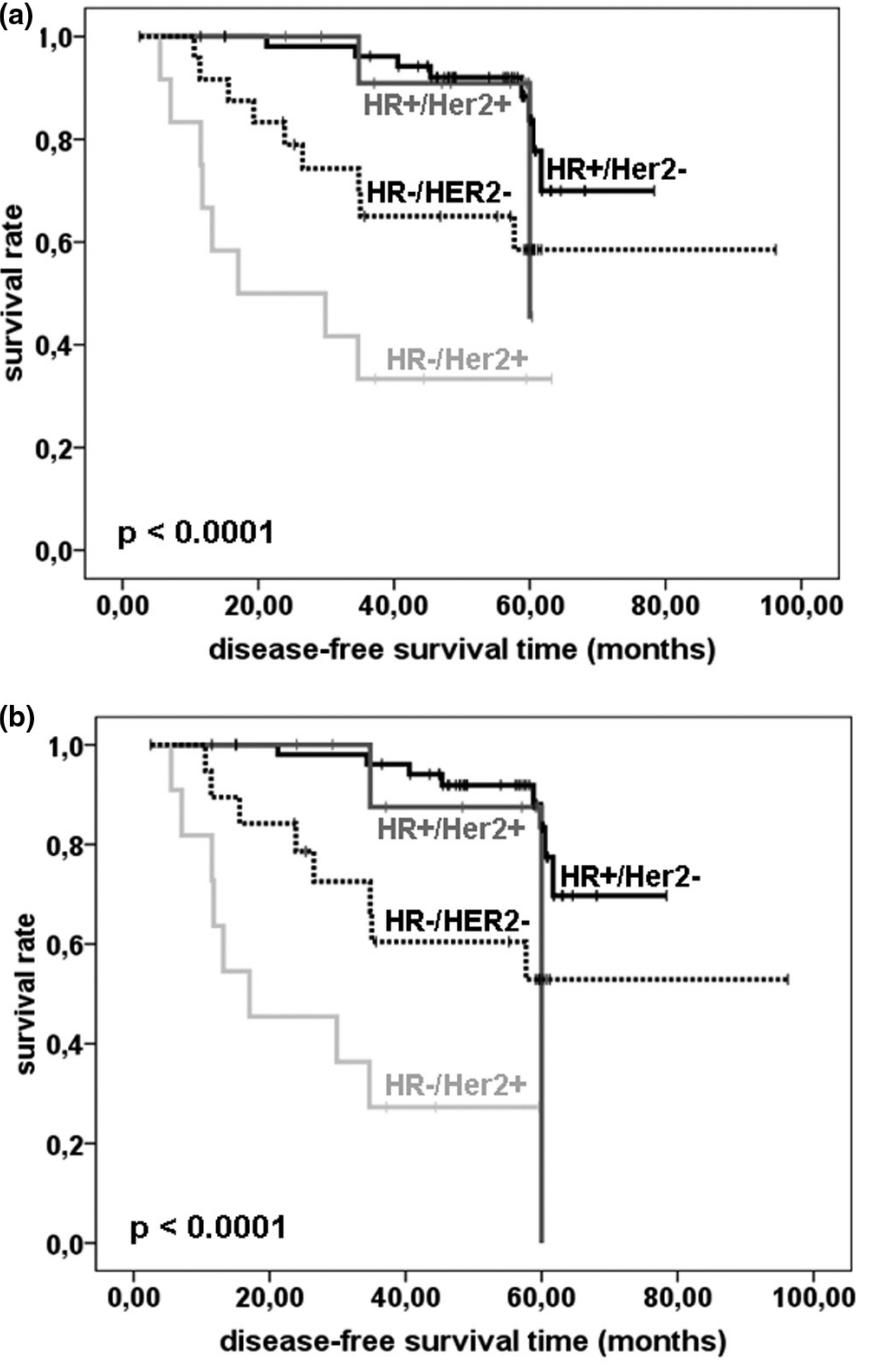
**(a)** Kaplan-Meier curve indicating disease-free survival times of patients in dependence of breast cancer subclassification. **(b) **Disease-free survival of patients who did not achieve a pCR in dependence of biology-based tumor type. p: Cox regression.

Patient prognosis was significantly dependent on the biology-based tumor type *P *< 0.0001, Figure 3A, Table 4): Interestingly, the behavior of HER2 expressing tumors was dependent on co-expression of HR. Thus, tumors from the HR+/HER2+ category had a relatively favorable prognosis similar to HR+/HER2- cancers (hazard ratio 1.26 compared to HR+/HER2-, *P *= 0.770; 3-year survival rate 90.0%). In contrast, a HR-/HER2- status was associated with a significant deterioration of the prognosis (hazard ratio 2.23 compared to HR+/HER2-; 3-year survival 65.0%, *P *= 0.016) and patients with HR-/HER2+ tumors had the shortest survival time of all groups (hazard ratio 9.32 compared to HR+/HER2-; 3-year survival 33.3%, *P *< 0.0001). Patients with HR+/HER2- tumors had the longest time to disease progression with a 3-year survival rate of 96.3%.

**Table 4:** Correlation with disease-free survival (Cox regression analysis)

	n	events	3-year survival rate (%)	hazard ratio	95% CI	p
**biology-based tumor type**	105	27				< 0.0001^a^
HR+/HER2-	56	8	96.3	1.00		
HR+/HER2+	13	2	90.0	1.26	0.27-5.98	0.770
HR-/HER2+	12	8	33.3	9.32	3.45-25.13	< 0.0001
HR-/HER2-	24	9	65.0	2.23	1.24-8.40	0.016
**Ki67**	96	21				
<= 20%	79	16	87.7	1.00		
> 20%	17	5	70.6	1.43	0.52-3.94	0.486
**pCR**	105	27				
no pCR	95	26	80.0	1.00		
pCR	10	1	90.0	0.29	0.04-2.15	0.227
**ypN**	105	27				
ypN0	65	13	83.1	1.00		
ypN1-2	40	14	52.4	2.46	1.142-5.302	0.021

CI: confidence interval.

^a^: overall significance.

Analyzing only the 95 patients without pCR we found the same results, especially women with HR+/HER2- and HR+/HER2+ tumors relapsed in only 10 from 55 cases (*P *< 0.0001, log rank test, 18.2%, Figure 3B). In contrast, women with HR-/HER2+ cancers relapsed frequently, in 8 out of 11 cases (72.7%) as did women with HR-/HER2- tumors (8/19, 42.1%).

### Association of other factors with DFS

Pathological lymph node state after chemotherapy (ypN) was a further significant prognostic factor for DFS (*P *= 0.021, Table 4). No other clinico-pathological factor or biomarker, including Ki67 labeling index, had prognostic impact in the whole study group or in the HR-/HER2- subgroup (data not shown).

### Association with DFS - multivariate analysis

An exploratory multivariate Cox regression analysis including biology-based tumor types, nodal status and pCR (Table 2) confirmed biology-based tumor type as an independent prognostic factor for DFS. Thus, the HR-/HER2+ as well as the HR-/HER2- subgroup remained significant risk factors for a disease relapse as compared to HR+/HER2- tumors (*P *< 0.0001 and 0.003, respectively). Nodal state remained a significant prognostic factor in multivariate analysis, too (*P *= 0.013).

## Discussion

Our study demonstrates that breast cancer subclassification based on HR and HER2 expression as used in standard diagnostics bears potential for the prediction of a pCR in patients with operable breast cancer receiving neoadjuvant chemotherapy with anthracycline and taxane and has a prognostic impact. Interestingly, the coexpression of HER2 and HR was found to be relevant for prediction of therapy response as well as assessment of long term benefit. HR+/HER2+ co-expressing tumors had a high response rate and showed a favorable DFS similarly to HR+/HER2- tumors, which however, responded rarely. A particularly low response rate as well as a poor prognosis was seen for HR-/HER2+ breast cancers. Further on, HR-/HER2- tumors were linked to a higher pCR rate, yet relapsed significantly earlier if they did not achieve a pCR.

Even though the determination of HR and HER2 status is routinely performed in breast cancer diagnostics, the predictive and prognostic value of a classification based explicitly on HR/HER2 expression has been rarely analyzed and in part only been reported in supplemental data. Basically, these studies showed similar results to ours: Guarneri et al. found retrospectively in a cohort of 1,731 breast cancer patients treated with varying neoadjuvant anthracycline-based regimes that ER+/HER2+ carcinomas had a higher pCR rate than ER+/HER2- tumors (15.3% vs. 6%) [30]. The highest pCR rates in this study were observed in ER-/HER2+ (29%) and in ER-/HER2- carcinomas (22.4%). Five-year disease-free survival rate was only slightly lower in ER+/HER2+ than in ER+/HER2- tumors (66.3% vs. 60.2%) and was lowest in ER-/HER2+ tumors (43.7%) just like in our study. Similarly, in a retrospective study including 1,118 patients treated with various neoadjuvant chemotherapeutic regimes Liedke et al. found equally high pCR rates in ER+/HER2+ and ER-/HER2- carcinomas (21% vs. 22%; ER+/HER2-: 5%, ER-/HER2+: 31%). Three-year disease-free survival rate was similar in ER+/HER2+ and in ER+/HER2- carcinomas (70% vs. 73%) and was as low in ER-/HER2+ tumors as in ER-/HER2- tumors (61% and 63%) [31]. In a study setting similar to ours, Carey et al. showed in 107 patients treated with anthracycline-based neoadjuvant chemotherapy that in HR+/HER2+ tumors pCR rate was higher than in HR+/HER2- tumors, and DFS was worse in HR+/HER2+ co-expressing than in HR+/HER2- tumors, yet was still clearly better than in HR-/HER2- or HR-/HER2+ tumors [32]. Regarding these results and our data presented here, it is conceivable that patients with HR and HER2 co-expressing breast carcinomas might constitute a group that particularly benefits from neoadjuvant chemotherapy as demonstrated by high pCR rates and favorable survival times. This is in contrast to HR+/HER2- tumors that rarely respond but nevertheless show favorable survival rates. The results according to the response rate of HR-/HER2+ tumors are conflictive as the studies cited above found high pCR rates in this subgroup, in contrast to our results. Of note, neither in the Geparduo cohort nor in the cohorts described above trastuzumab had been included in the neoadjuvant therapy regime as this was no standard at the time of study execution. As newer studies using trastuzumab in patients with HER2+ tumors have shown almost doubled pCR rates, it will be compelling to elucidate whether the predictive and prognostic effects described above would be altered by the addition of anti-HER2 agents.

Some interesting facts about the molecular interaction of hormone receptors (particularly ER) and HER2 have been reported to date: estrogen generally downregulates HER2 expression [33,34], a mechanism that does not seem to be relevant in HR/HER2 co-expressing carcinomas from hitherto unknown reasons [16]. Moreover, ER can activate HER2 by membrane non-genomic estrogen signaling, while HER2 activates ligand-independently ER by mitogen-activated protein kinase (MAPK)-/protein kinase B (AKT)-mediated phosphorylation [35]. These interactions have been supposed to be underlying the relative resistance and worse prognosis of breast cancers that co-express ER and HER2 and that have been treated with tamoxifen [36]. However, the situation in patients that are treated with primary chemotherapy is presumably quite different and there are no functional data that explicitly refer to this group of tumors. In the adjuvant setting HER2+ tumors respond well to an anthracycline-based therapy [37] and it is conceivable that this might be also the case in HR+/HER2+ tumors in the neoadjuvant setting as reflected by high pCR rates. For long-term prognosis the phenotype of HR positive tumors cells (higher differentiation, slower proliferation, etc.) seems to be more relevant in receptor co-expressing tumors and may even be amplified by HER2-mediated ER activation. Yet, the exact mechanisms remain to be elucidated in future functional studies.

The other biology-based tumor type for which we found a particular behavior as to response and survival is the HR-/HER2- (triple negative) subgroup. In line with previous reports in the neoadjuvant and adjuvant setting [30-32,38-40], we observed a relatively short survival time in spite of a high response rate. Carey and Liedke explained this with the exceptionally poor prognosis of patients with HR-/HER2- tumors not achieving a pCR [31,32]. We also saw a higher rate of disease-relapses in HR-/HER2- tumors without pCR than with pCR (42.1% vs. 20%). A predictive factor for neoadjuvant chemotherapy response might thus also be a reliable prognostic factor in the HR-/HER2- subtype. However, in our group highly proliferating tumors were more likely to respond, which may be explained by the fact that actively dividing cells are the target of cytotoxic drugs, but a high Ki67 labeling index was not linked to a better prognosis. Independent of the biology-based tumor type, Ki67 staining *per se *was an independent predictive but no prognostic factor in the whole study group, indicating that as to long-term survival proliferation is not as relevant as HR or HER2 expression. Yet, our findings support the concept that HR-/HER2- carcinomas are a heterogeneous groups of tumors which should be subdivided further [18,19]. The expression of basal cytokeratins has been reported as one distinctive feature of the so-called basal-like carcinomas, a highly aggressive breast cancer subtype according to the concept of intrinsic breast cancer subtypes, which have been defined by gene expression analysis [12]. In our whole study group CK5/6 expression was a predictive factor, but within the HR-/HER2- group the addition of CK5/6 expression did not add predictive or prognostic information to the determination of ER, PgR, and HER2, which however might have been a sample size problem. The problem with the poor-prognosis HR-/HER2- tumors is that no specific targeted therapy exists to date, in contrast to endocrine therapy in HR+ and trastuzumab or lapatinib in HER2+ breast cancers. Our hypothesis of a special predictive and prognostic role of COX-2 or YB-1-expression (for both molecules targeted therapies are available or are in development) [41,42] in HR-/HER2- carcinomas could not be proven in our study.

Certain limitations of our study should be stated: Due to the retrospective evaluation and the limited sample size it is primarily a hypothesis-generating study, and results remain to be investigated further in larger cohorts, preferentially in prospective trials [43]. However, the setting of a clinical trial ensures a clearly described population, homogenous treatment as well as well-documented and monitored data.

## Conclusions

In summary, our results demonstrate that a breast cancer classification, simply based on the expression of the standard markers ER, PgR, and HER2 bears independent predictive and prognostic potential. Patients with HR-/HER2- tumors, particularly those without achievement of a pCR, are in need for further treatment options. Patients with HR-/HER+ tumors had an unfavorable prognosis, but can now be treated with anti-HER2 agents. In contrast, for HR+/HER2- carcinomas pCR was not relevant for prognosis, as DFS was long in spite of a low response rate. The HR+/HER2+ co-expressing carcinomas, so far insufficiently investigated, emerged as a group of tumors with a good response rate to neoadjuvant anthracycline/taxane chemotherapy and a favorable prognosis. This interesting group of tumors should be further investigated in prospective clinical trials and in functional studies.

## Abbreviations

AC-DOC: doxorubicin plus cyclophosphamide followed by docetaxel; CI: confidence interval; CK5/6: cytokeratin 5/6; COX-2: cyclooxigenase-2; ddADOC: dose-dense doxorubicin plus docetaxel; DFS: disease-free survival; ER: estrogen receptor; HR: hormone receptor; HR+/HER2-: hormone receptor positive/HER2 negative; HR+/HER2+: hormone receptor positive/HER2 positive; HR-/HER2+: hormone receptor negative/HER2 positive; HR-/HER2-: hormone receptor negative/HER2 negative; OR: odds ratio; pCR: pathological complete response; PgR: progesterone receptor; SISH: silver-enhanced in situ hybridization; TMA: tissue microarray; YB-1: Y-box binding protein 1.

## Competing interests

The authors declare that they have no competing interests.

## Authors' contributions

SDE evaluated the immunohistochemical and SISH stainings, performed statistical analysis, contributed to the study design, and drafted the manuscript. SL, BM, CD, and GVM participated in data interpretation and study design, and extensively reviewed the manuscript. MR and MK participated in data acquisition and interpretation. JB participated in the statistical analysis. KS was responsible for the development of the software for evaluation of the TMA. AN, WW, and MD participated in data interpretation and extensively reviewed the manuscript. JUB, BG, ADB, CJ, MK, and KR participated in the clinical studies as well as the collection of tumor samples. All authors read and approved the final manuscript.

## Supplementary Material

Additional file 1 A table listing the clinico-pathological characteristics of the study group. Click here for file

## References

[B1] Goldhirsch A Wood WC Gelber RD Coates AS Thürlimann B Senn HJ Progress and promise: highlights of the international expert consensus on the primary therapy of early breast cancer 2007 Ann Oncol 2007 18 1133 44 10.1093/annonc/mdm271 17675394

[B2] Kaufmann M Hortobagyi GN Goldhirsch A Scholl S Makris A Valagussa P Blohmer JU Eiermann W Jackesz R Jonat W Lebeau A Loibl S Miller W Seeber S Semiglazov V Smith R Souchon R Stearns V Untch M von Minckwitz G Recommendations from an international expert panel on the use of neoadjuvant (primary) systemic treatment of operable breast cancer: an update J Clin Oncol 2006 24 1940 9 10.1200/JCO.2005.02.6187 16622270

[B3] Fisher B Brown A Mamounas E Wieand S Robidoux A Margolese RG Cruz AB Jr Fisher ER Wickerham DL Wolmark N DeCillis A Hoehn JL Lees AW Dimitrov NV Effect of preoperative chemotherapy on local-regional disease in women with operable breast cancer: findings from National Surgical Adjuvant Breast and Bowel Project B-18 J Clin Oncol 1997 15 2483 93 9215816 10.1200/JCO.1997.15.7.2483

[B4] Fisher B Bryant J Wolmark NE Brown A Fisher ER Wickerham DL Begovic M DeCillis A Robidoux A Margolese RG Cruz AB Jr Hoehn JL Lees AW Dimitrov NV Bear HD Effect of preoperative chemotherapy on the outcome of women with operable breast cancer J Clin Oncol 1998 16 2672 85 9704717 10.1200/JCO.1998.16.8.2672

[B5] Scholl SM Pierga JY Asselain B Beuzeboc P Dorval T Garcia-Giralt E Jouve M Palangié T Remvikos Y Durand JC Breast tumour response to primary chemotherapy predicts local and distant control as well as survival Eur J Cancer 1995 31A 1969 75 10.1016/0959-8049(95)00454-8 8562150

[B6] Kuerer HM Newman LA Smith TL Ames FC Hunt KK Dhingra K Theriault RL Singh G Binkley SM Sneige N Buchholz TA Ross MI McNeese MD Buzdar AU Hortobagyi GN Singletary SE Clinical course of breast cancer patients with complete pathologic primary tumor and axillary lymph node response to doxorubicin-based neoadjuvant chemotherapy J Clin Oncol 1999 17 460 9 10080586 10.1200/JCO.1999.17.2.460

[B7] Hage JA van der Velde CJ van de Julien JP Tubiana-Hulin M Vandervelden C Duchateau L Preoperative chemotherapy in primary operable breast cancer: results from the European Organization for Research and Treatment of Cancer trial 10902 J Clin Oncol 2001 19 4224 37 11709566 10.1200/JCO.2001.19.22.4224

[B8] Gonzalez-Angulo AM McGuire SE Buchholz TA Tucker SL Kuerer HM Rouzier R Kau SW Huang EH Morandi P Ocana A Cristofanilli M Valero V Buzdar AU Hortobagyi GN Factors predictive of distant metastases in patients with breast cancer who have a pathologic complete response after neoadjuvant chemotherapy J Clin Oncol 2005 23 7098 104 10.1200/JCO.2005.11.124 16192593

[B9] Kaufmann M von Minckwitz G Rody A Preoperative (neoadjuvant) systemic treatment of breast cancer Breast 2005 14 576 81 10.1016/j.breast.2005.08.010 16199160

[B10] Bear HD Anderson S Brown A Smith R Mamounas EP Fisher B Margolese R Theoret H Soran A Wickerham DL Wolmark N The effect on tumor response of adding sequential preoperative docetaxel to preoperative doxorubicin and cyclophosphamide: preliminary results from National Surgical Adjuvant Breast and Bowel Project Protocol B-27 J Clin Oncol 2003 21 4165 74 10.1200/JCO.2003.12.005 14559892

[B11] Untch M Konency G Ditsch N Sorokina Y Moebus V Muck B Kuhn W Bastert G Werner Ch Thomssen Ch Wallwiener D Albert U Bothmann G Kreienberg R Lück HJ Dose-dense sequential epirubicin-paclitaxel as preoperative treatment of breast cancer: Results of a randomized AGO study [abstract] Proc Am Soc Clin Oncol 2002 21 34a

[B12] Sørlie T Perou CM Tibshirani R Aas T Geisler S Johnsen H Hastie T Eisen MB Rijn M van de Jeffrey SS Thorsen T Quist H Matese JC Brown PO Botstein D Eystein Lønning P Børresen-Dale AL Gene expression patterns of breast carcinomas distinguish tumor subclasses with clinical implications Proc Natl Acad Sci USA 2001 98 10869 74 10.1073/pnas.191367098 11553815 PMC58566

[B13] Calza S Hall P Auer G Bjöhle J Klaar S Kronenwett U Liu ET Miller L Ploner A Smeds J Bergh J Pawitan Y Intrinsic molecular signature of breast cancer in a population-based cohort of 412 patients Breast Cancer Res 2006 8 R34 10.1186/bcr1517 16846532 PMC1779468

[B14] Sorlie T Tibshirani R Parker J Repeated observation of breast tumor subtypes in independent gene expression data sets Proc Natl Acad Sci USA 2003 100 8418 23 10.1073/pnas.0932692100 12829800 PMC166244

[B15] Sørlie T Wang Y Xiao C Johnsen H Naume B Samaha RR Børresen-Dale AL Distinct molecular mechanisms underlying clinically relevant subtypes of breast cancer: gene expression analyses across three different platforms BMC Genomics 2006 7 127 10.1186/1471-2164-7-127 16729877 PMC1489944

[B16] Ciocca DR Gago FE Fanelli MA Calderwood SK Co-expression of steroid receptors (estrogen receptor alpha and/or progesterone receptors) and Her-2/neu: Clinical implications J Steroid Biochem Mol Biol 2006 102 32 40 10.1016/j.jsbmb.2006.09.008 17049840

[B17] Prat A Baselga J The role of hormonal therapy in the management of hormonal-receptor-positive breast cancer with co-expression of HER2 Nat Clin Pract Oncol 2008 5 531 42 10.1038/ncponc1179 18607391

[B18] Irvin WJ Jr Carey LA What is triple-negative breast cancer? Eur J Cancer 2008 44 2799 805 10.1016/j.ejca.2008.09.034 19008097

[B19] Rakha EA Tan DS Foulkes WD Ellis IO Tutt A Nielsen TO Reis-Filho JS Are triple-negative tumours and basal-like breast cancer synonymous? Breast Cancer Res 2007 9 404 10.1186/bcr1827 18279542 PMC2246182

[B20] Denkert C Winzer KJ Müller BM Weichert W Pest S Köbel M Kristiansen G Reles A Siegert A Guski H Hauptmann S Elevated expression of cyclooxygenase-2 is a negative prognostic factor for disease free survival and overall survival in patients with breast carcinoma Cancer 2003 97 2978 87 10.1002/cncr.11437 12784332

[B21] Janz M Harbeck N Dettmar P Berger U Schmidt A Jürchott K Schmitt M Royer HD Y-box factor YB-1 predicts drug resistance and patient outcome in breast cancer independent of clinically relevant tumor biologic factors HER2, uPA and PAI-1 Int J Cancer 2002 97 278 82 10.1002/ijc.1610 11774277

[B22] von Minckwitz G Raab G Caputo A Schütte M Hilfrich J Blohmer JU Gerber B Costa SD Merkle E Eidtmann H Lampe D Jackisch C du Bois A Kaufmann M Doxorubicin with cyclophosphamide followed by docetaxel every 21 days compared with doxorubicin and docetaxel every 14 days as preoperative treatment in operable breast cancer: the GEPARDUO study of the German Breast Group J Clin Oncol 2005 23 2676 85 10.1200/JCO.2005.05.078 15837982

[B23] Elston CW Ellis IO Pathological prognostic factors in breast cancer. I. The value of histological grade in breast cancer: experience from a large study with long-term follow-up Histopathology 1991 19 403 10 10.1111/j.1365-2559.1991.tb00229.x 1757079

[B24] Wolff AC Hammond ME Schwartz JN Hagerty KL Allred DC Cote RJ Dowsett M Fitzgibbons PL Hanna WM Langer A McShane LM Paik S Pegram MD Perez EA Press MF Rhodes A Sturgeon C Taube SE Tubbs R Vance GH Vijver M van de Wheeler TM Hayes DF American Society of Clinical Oncology/College of American Pathologists guideline recommendations for human epidermal growth factor receptor 2 testing in breast cancer J Clin Oncol 2007 25 118 45 10.1200/JCO.2006.09.2775 17159189

[B25] Petit T Wilt M Velten M Millon R Rodier JF Borel C Mors R Haegelé P Eber M Ghnassia JP Comparative value of tumour grade, hormonal receptors, Ki-67, HER-2 and topoisomerase II alpha status as predictive markers in breast cancer patients treated with neoadjuvant anthracycline-based chemotherapy Eur J Cancer 2004 40 205 11 10.1016/S0959-8049(03)00675-0 14728934

[B26] Fujii T Kawahara A Basaki Y Hattori S Nakashima K Nakano K Shirouzu K Kohno K Yanagawa T Yamana H Nishio K Ono M Kuwano M Kage M Expression of HER2 and estrogen receptor alpha depends upon nuclear localization of Y-box binding protein-1 in human breast cancers Cancer Res 2008 68 1504 12 10.1158/0008-5472.CAN-07-2362 18316615

[B27] Wu J Lee C Yokom D Jiang H Cheang MC Yorida E Turbin D Berquin IM Mertens PR Iftner T Gilks CB Dunn SE Disruption of the Y-box binding protein-1 results in suppression of the epidermal growth factor receptor and HER-2 Cancer Res 2006 66 4872 9 10.1158/0008-5472.CAN-05-3561 16651443

[B28] Nielsen TO Hsu FD Jensen K Cheang M Karaca G Hu Z Hernandez-Boussard T Livasy C Cowan D Dressler L Akslen LA Ragaz J Gown AM Gilks CB Rijn M van de Perou CM Immunohistochemical and clinical characterization of the basal-like subtype of invasive breast carcinoma Clin Cancer Res 2004 10 5367 74 10.1158/1078-0432.CCR-04-0220 15328174

[B29] Abd El-Rehim DM Ball G Pinder SE Rakha E Paish C Robertson JF Macmillan D Blamey RW Ellis IO High-throughput protein expression analysis using tissue microarray technology of a large well-characterised series identifies biologically distinct classes of breast cancer confirming recent cDNA expression analyses Int J Cancer 2005 116 340 50 10.1002/ijc.21004 15818618

[B30] Guarneri V Broglio K Kau SW Cristofanilli M Buzdar AU Valero V Buchholz T Meric F Middleton L Hortobagyi GN Gonzalez-Angulo AM Prognostic value of pathologic complete response after primary chemotherapy in relation to hormone receptor status and other factors J Clin Oncol 2006 24 1037 44 10.1200/JCO.2005.02.6914 16505422

[B31] Liedtke C Mazouni C Hess KR André F Tordai A Mejia JA Symmans WF Gonzalez-Angulo AM Hennessy B Green M Cristofanilli M Hortobagyi GN Pusztai L Response to neoadjuvant therapy and long-term survival in patients with triple-negative breast cancer J Clin Oncol 2008 26 1275 81 10.1200/JCO.2007.14.4147 18250347

[B32] Carey LA Dees EC Sawyer L Gatti L Moore DT Collichio F Ollila DW Sartor CI Graham ML Perou CM The triple negative paradox: primary tumor chemosensitivity of breast cancer subtypes Clin Cancer Res 2007 13 2329 34 10.1158/1078-0432.CCR-06-1109 17438091

[B33] Read LD Keith D Jr Slamon DJ Katzenellenbogen BS Hormonal modulation of HER-2/neu protooncogene messenger ribonucleic acid and p185 protein expression in human breast cancer cell lines Cancer Res 1990 50 3947 51 1972345

[B34] De Bortoli M Dati C Antoniotti S Maggiora P Sapei ML Hormonal regulation of c-erbB-2 oncogene expression in breast cancer cells J Steroid Biochem Mol Biol 1992 43 21 5 10.1016/0960-0760(92)90183-J 1356014

[B35] Ali S Metzger D Bornert JM Chambon P Modulation of transcriptional activation by ligand-dependent phosphorylation of the human oestrogen receptor A/B region EMBO J 1993 12 1153 60 8458328 10.1002/j.1460-2075.1993.tb05756.x PMC413317

[B36] Gago FE Fanelli MA Ciocca DR Co-expression of steroid hormone receptors (estrogen receptor alpha and/or progesterone receptors) and Her2/neu (c-erbB-2) in breast cancer: clinical outcome following tamoxifen-based adjuvant therapy J Steroid Biochem Mol Biol 2006 98 36 40 10.1016/j.jsbmb.2005.07.002 16188438

[B37] Rayson D Richel D Chia S Jackisch C Vegt S van der Suter T Anthracycline-trastuzumab regimens for HER2/neu-overexpressing breast cancer: current experience and future strategies Ann Oncol 2008 19 1530 9 10.1093/annonc/mdn292 18480068

[B38] Banerjee S Reis-Filho JS Ashley S Steele D Ashworth A Lakhani SR Smith IE Basal-like breast carcinomas: clinical outcome and response to chemotherapy J Clin Pathol 2006 59 729 35 10.1136/jcp.2005.033043 16556664 PMC1860434

[B39] Diallo-Danebrock R Ting E Gluz O Herr A Mohrmann S Geddert H Rody A Schaefer KL Baldus SE Hartmann A Wild PJ Burson M Gabbert HE Nitz U Poremba C Protein expression profiling in high-risk breast cancer patients treated with high-dose or conventional dose-dense chemotherapy Clin Cancer Res 2007 13 488 97 10.1158/1078-0432.CCR-06-1842 17255270

[B40] Rodríguez-Pinilla SM Sarrió D Honrado E Hardisson D Calero F Benitez J Palacios J Prognostic significance of basal-like phenotype and fascin expression in node-negative invasive breast carcinomas Clin Cancer Res 2006 12 1533 9 10.1158/1078-0432.CCR-05-2281 16533778

[B41] Howe LR Inflammation and breast cancer. Cyclooxygenase/prostaglandin signaling and breast cancer Breast Cancer Res 2007 9 210 10.1186/bcr1678 17640394 PMC2206709

[B42] Fujii T Yokoyama G Takahashi H Toh U Kage M Ono M Shirouzu K Kuwano M Preclinical and clinical studies of novel breast cancer drugs targeting molecules involved in protein kinase C signaling, the putative metastasis-suppressor gene Cap43 and the Y-box binding protein-1 Curr Med Chem 2008 15 528 37 10.2174/092986708783769759 18336267

[B43] von Minckwitz G Kaufmann M Kümmel S Fasching P Eiermann W Blohmer J-U Costa SD Sibylle L Dietmar V Untch M Integrated meta-analysis on 6402 patients with early breast cancer receiving neoadjuvant anthracycline-taxane +/- trastuzumab containing chemotherapy [abstract] San Antonio Breast Cancer Symposium 2008

