# Supplementary material for: Identification of biology-based breast cancer types with distinct predictive and prognostic features: role of steroid hormone and HER2 receptor expression in patients treated with neoadjuvant anthracycline/taxane-based chemotherapy
Source: Breast Cancer Res. 2009 Sep 16;11(5):R69. doi: 10.1186/bcr2363 (PMC2790846; doi:10.1186/bcr2363)
Supplement: Additional file 1 — A table listing the clinico-pathological characteristics of the study group. [file bcr2363-S1.DOC]

**Additional data file 1 Clinico-pathological characteristics of the study group**

| **parameter** | **no of patients (%)** |
| --- | --- |
| **all** | 116 (100%) |
| **age**  median (range) | 50 (27-71) |
| **cT**  cT1  cT2  cT3 | 9 (7.8%)  87 (75.0%)  20 (17.2%) |
| **cN**  cN0  cN1  cN2 | 79 (68.1%)  34 (29.3%)  3 ( 2.6%) |
| **histology**  ductal invasive  lobular  others | 98 (84.5%)  14 (12.1%)  4 ( 3.4%) |
| **grade**  G1  G2  G3 | 14 (12.1%)  62 (53.4%)  40 (34.5%) |
| **pCR**  no pCR  pCR | 103 (88.8%)  13 (11.2%) |
| **therapy arm**  ddADOC  AC-DOC | 61 (52.6%)  55 (47.4%) |
